# The Conformational Equilibrium of the Neuropeptide Y2 Receptor in Bilayer Membranes

**DOI:** 10.1002/anie.202006075

**Published:** 2020-09-30

**Authors:** Ulrike Krug, Anika Gloge, Peter Schmidt, Johanna Becker‐Baldus, Frank Bernhard, Anette Kaiser, Cindy Montag, Marcel Gauglitz, Sergey A. Vishnivetskiy, Vsevolod V. Gurevich, Annette G. Beck‐Sickinger, Clemens Glaubitz, Daniel Huster

**Affiliations:** ^1^ Institute of Medical Physics and Biophysics University of Leipzig Härtelstr. 16–18 04107 Leipzig Germany; ^2^ Institute of Biophysical Chemistry Goethe University Frankfurt Germany; ^3^ Center for Biomolecular Magnetic Resonance Goethe University Frankfurt Germany; ^4^ Institute of Biochemistry University of Leipzig Leipzig Germany; ^5^ Berlin Joint Electron Paramagnetic Resonance Laboratory Free University Berlin Germany; ^6^ Department of Pharmacology Vanderbilt University Nashville TN USA

**Keywords:** arrestin, molecular switch, NMR spectroscopy, receptors, structural dynamics

## Abstract

Dynamic structural transitions within the seven‐transmembrane bundle represent the mechanism by which G‐protein‐coupled receptors convert an extracellular chemical signal into an intracellular biological function. Here, the conformational dynamics of the neuropeptide Y receptor type 2 (Y2R) during activation was investigated. The apo, full agonist‐, and arrestin‐bound states of Y2R were prepared by cell‐free expression, functional refolding, and reconstitution into lipid membranes. To study conformational transitions between these states, all six tryptophans of Y2R were ^13^C‐labeled. NMR‐signal assignment was achieved by dynamic‐nuclear‐polarization enhancement and the individual functional states of the receptor were characterized by monitoring ^13^C NMR chemical shifts. Activation of Y2R is mediated by molecular switches involving the toggle switch residue Trp281^6.48^ of the highly conserved SWLP motif and Trp327^7.55^ adjacent to the NPxxY motif. Furthermore, a conformationally preserved “cysteine lock”‐Trp116^23.50^ was identified.

## Graphical abstract

Structural transitions of crucial molecular switches of the activation of the human neuropeptide Y receptor type 2 are reported from the ^13^C‐tryptophan labeled molecule by solid‐state NMR spectroscopy in lipid membranes. The molecule shows high structural dynamics in the apo and agonist‐bound states, which is reduced by arrestin binding.

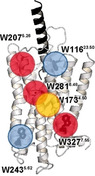

## Introduction

G protein‐coupled receptors (GPCRs) are ubiquitously found in humans and represent important pharmacological targets.^[1]^ Conformational flexibility represents a hallmark of GPCR function while their characteristic structural motif is the seven‐transmembrane (TM) helix bundle. Binding of an extracellular ligand induces a cascade of dynamic structural rearrangements within the TM region, especially a characteristic outwards movement of TM6,^[2]^ representing the initial signal transduction events. For the molecular understanding of the biological function of GPCRs, detailed characterization of the conformational transitions during activation is essential. As of today, structural data of 67 GPCRs (https://zhanglab.ccmb.med.umich.edu/GPCR‐EXP/) determined in the inactive, intermediate or active conformations are available. While the structural basis for the interaction of GPCRs with all G‐protein subtypes is becoming more complete, the dynamic processes that underlie the mechanisms by which agonist‐activated GPCRs interact with intracellular arrestins is still fragmentary. Arrestin is an important intracellular interaction partner of GPCRs that desensitizes G‐protein‐mediated signaling and creates the signal for receptor internalization.^[3]^ Until recently, structural data was only available for the complex of bovine (rhod)opsin with arrestin.[^4^, ^5^] Now, cryo‐EM structures of the neurotensin receptor 1,^[6]^ the β_1_ adrenoceptor,^[7]^ and the muscarinic M2 receptor^[8]^ in complex with β‐arrestin 1 (also known as arrestin‐2) have become available.

To fully understand the biological function of GPCRs in interaction with intracellular effectors, monitoring the conformational dynamics of specific sites by biophysical spectroscopy is most promising. Magnetic resonance methods such as EPR and NMR have been of key importance in this regard.[^2^, ^9^] Recently, DEER spectroscopy was used to map conformational changes of angiotensin II receptor using spin labels.^[10]^ This study revealed that different ligands induce diverse conformational signatures. In NMR spectroscopy, either native probes such as the methyl group of methionine[^11^, ^12^] and the indole ring of native or artificially introduced tryptophan (Trp) residues[^13^, ^14^, ^15^] or artificial fluorine labels attached to free cysteines[^16^, ^17^] have been used to describe the individual structural states of GPCRs during activation. The detected NMR chemical shift of a given site depends on the local chemical environment and protein structure, thus, alterations in NMR peak position indicate structural modifications in the receptor in response to agonist or G‐protein binding.^[18]^ Various NMR studies conducted in the absence and presence of ligands as well as intracellular binding partners such as G‐proteins, nano‐ or antibodies showed that GPCRs assume distinct conformations characteristic for the individual states.^[18]^ While agonist binding appears to destabilize the inactive state, it is not sufficient to stabilize the fully active receptor conformation.^[12]^

The current model of GPCR activation describes a number of well conserved residues that are crucial for the structural rearrangements of the protein upon activation, referred to as molecular switches.^[19]^ Using spectroscopic tools, the structural rearrangements of these residues can be studied in detail to understand the activation mechanisms of a GPCR for which the crystal structures of all substrates are not available. Here, we target the neuropeptide Y2 receptor (Y2R) and describe the conformational responses of specific sites of the molecule to ligand‐ and arrestin‐3 (also known as β‐arrestin 2) binding. Y2R is involved in the control of food intake and memory retention, mood disorders and epilepsy.^[20]^ Its natural ligand is the 36 residue hormone neuropeptide Y (NPY) that forms an amphipathic C‐terminal α‐helix.^[21]^ Upon binding to Y1R^[22]^ and Y2R,^[23]^ unwinding of the C‐terminal residues was detected allowing the agonist to reach deep into the binding pocket. On the intracellular side, Y2R couples to the inhibitory family of G‐proteins (G_i/o_)^[24]^ and has also been shown to recruit arrestin‐3.^[25]^ Furthermore, Y2R in membranes was characterized as a highly mobile molecule in solid‐state NMR experiments but without site‐resolution.[^26^, ^27^]

**Figure 1:**
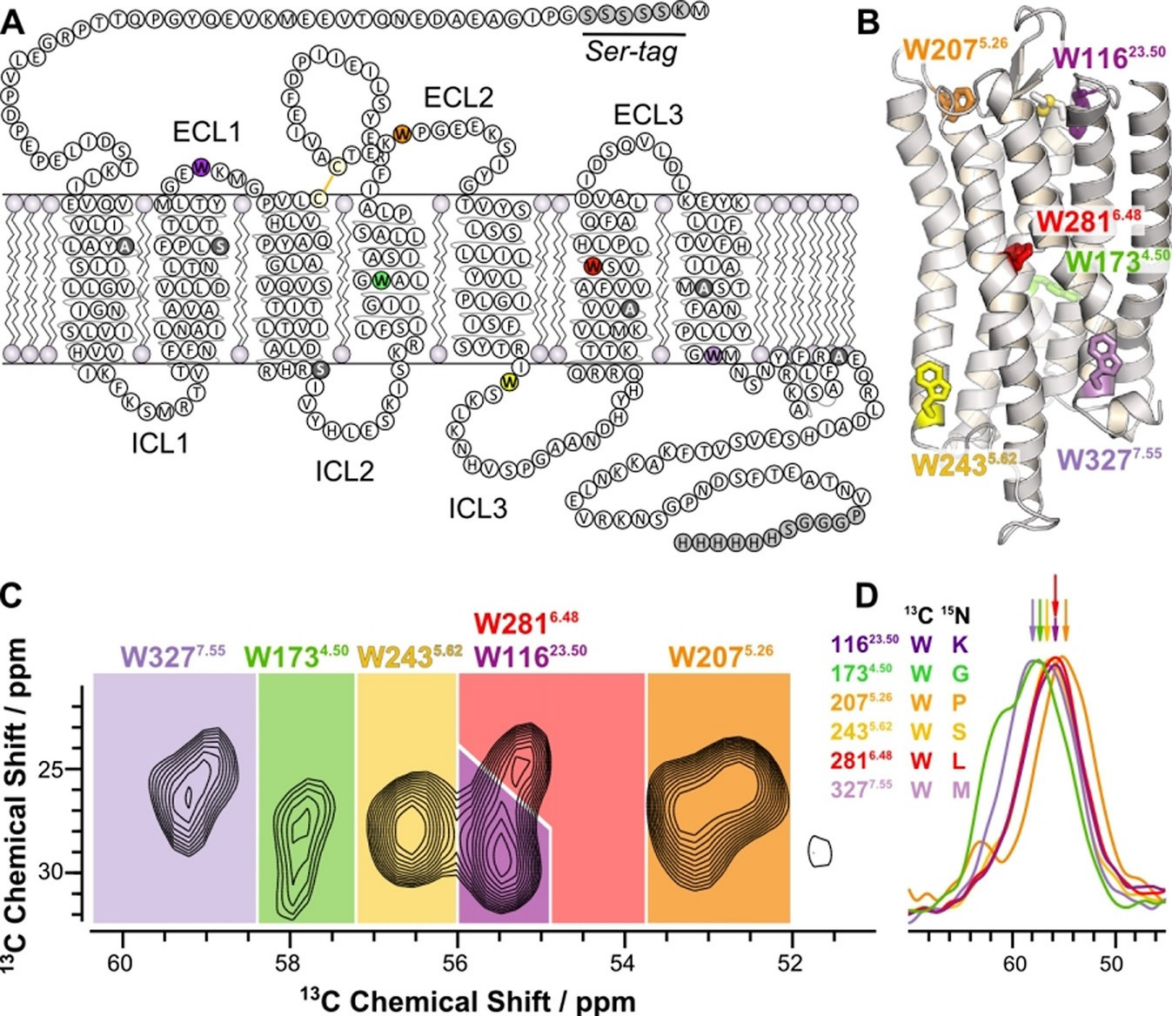
Sequence and structural architecture of Y2R and assignment of the ^13^Cα‐Trp signals of Y2R in DMPC membranes. A) Snake plot showing the Y2R construct used in this study with artificial amino acid insertions at the N‐ and C‐termini (gray). Cysteines not involved in disulfide bond formation were replaced by Ala or Ser (dark gray). The six ^13^C labeled Trp are colored. B) Structural model of Y2R^[23]^ with the Trp residues represented as stick models. C) Cα/Cβ region of a ^13^C‐^13^C DARR NMR spectrum of Y2R recorded at a temperature of −30 °C with the assignment to the specific residues. D) Cα region of the DNP NCOCX spectra used for signal assignment. NMR spectra were recorded using DNP for signal enhancement at a temperature of −164 °C. The labeling scheme for the sample preparation indicating the ^13^C‐labeled Trp and its ^15^N‐labeled successor amino acid is given. See also Supplementary Figure S2.

To provide a detailed site‐specific analysis of the dynamic properties of Y2R in the various functional states, native Trp residues represent valuable probes. In Y2R, there are only six native Trp, which significantly reduces the spectral complexity for NMR investigations. Using isotopically labeled native Trp residues as probes allows to (*i*) study the structure and dynamics of the Cα backbone with site resolution in (*ii*) a DMPC phospholipid membrane representing a very natural environment of GPCRs. The six Trp residues in Y2R are fairly equally distributed over the structure of Y2R (Figure 1) with two Trp in the extracellular loops (Trp116^23.50^, Trp207^5.26^, the superscript refers to the Ballesteros‐Weinstein nomenclature^[28]^), one in the center of TM4 (Trp173^4.50^), the “toggle switch” Trp281^6.48^ pointing into the ligand binding pocket, and two Trp on the intracellular side (Trp243^5.62^ and Trp327^7.55^). Thus, the six native Trp residues yield information from several key motifs of the receptor. By integration of cell‐free (CF) protein expression, dynamic nuclear polarization enhancement (DNP) and conventional NMR methods, specifically ^13^C‐labeled Y2R was prepared and the individual conformational changes of the different structural motifs of the Y2R in response to ligand and arrestin‐3 binding were analyzed.

## Results and Discussion

### Cell‐Free Expression, Reconstitution, and Pharmacology of Y2R

Y2R used in this study was prepared by CF expression in the precipitated form. For better expression yields, the construct contained short N‐ and C‐terminal amino acid insertions along with a His tag for purification (Figure 1). Six Cys residues not involved in disulfide bond formation were replaced by Ala or Ser to eliminate receptor aggregation during refolding.^[29]^ The term “Y2R” below refers to this construct. CF expression, purification and reconstitution of Y2R are detailed in the experimental section (Figure S1). Briefly, after CF expression, precipitated Y2R was solubilized by SDS in the presence of DTT. Y2R was purified and the SDS concentration was reduced in the presence of a redox shuffling system to form the native disulfide bridge. Y2R was reconstituted in preformed DMPC/DHPC bicelles.^[30]^ By removal of DHPC, planar membranes were formed with a residual DHPC content of less than 5 to 10 %. The ligand binding capacity of the reconstituted receptor was shown by a fluorescence polarization assay^[31]^ and the *K*_D_ value for NPY binding was determined to be ≈35 nM (Figure S1). To prepare Y2R in different functional states, NPY as well as the phosphorylation‐independent arrestin‐3‐3A variant^[32]^ were added. Binding of arrestin‐3‐3A was confirmed by a pull‐down assay (Figure S1).

### NMR Investigation of the Trp Sites of Y2R

For the NMR experiments, all Trp residues were ^13^C‐labeled by adding U‐^13^C‐Trp to the CF reaction mix. Using dipolar assisted rotational resonance (DARR) NMR spectroscopy (10 ms mixing time) at −30 °C, all one bond correlations were observed. Most characteristic is the Cα/Cβ region of the DARR NMR spectrum with six relatively well resolved crosspeaks at chemical shifts between ≈53 and 59 ppm for the ^13^Cα and ≈24 to 31 ppm for the ^13^Cβ sites (Figure 1 C). To assign these signals, a mutagenesis approach as described in the literature[^13^, ^14^, ^33^] was first considered. Y2R mutants were prepared each with one Trp replaced by Phe or Thr. But no full unambiguous assignment could be extracted from these experiments due to unpredictable chemical shift changes. In alternative experiments, the ^13^C NMR signals from each Trp were assigned through correlations to the successive ^15^N‐labeled residue by NCOCX correlation spectra.^[34]^ To this end, six ^13^C‐Trp/^15^N‐X‐labeled Y2R variants were prepared using U‐^13^C‐Trp and ^15^N‐labeling of the successor residue type (X). Fortunately, the Y2R sequence contains six unique amino acids following Trp allowing for unambiguous assignment of the following pairs: ^13^C‐Trp116^23.50^/^15^N‐Lys117^23.51^, ^13^C‐Trp173^4.50^/ ^15^N‐Gly174^4.51^, ^13^C‐Trp207^5.26^/^15^N‐Pro208^5.27^, ^13^C‐Trp243^5.62^/^15^N‐Ser244^5.63^, ^13^C‐Trp281^6.48^/^15^N‐Leu282^6.49^, and ^13^C‐Trp327^7.55^/^15^N‐Met328^7.56^. To increase the sensitivity, NMR experiments were carried out using dynamic nuclear polarization (DNP)^[35]^ yielding enhancement factors between ≈40 and 70 (Figure S2). The six 1D NCOCX spectra were referenced internally using the signals of glycerol and superimposed providing the unique ^13^Cα chemical shifts for all Trp residues. The assigned Cα/Cβ region of the ^13^C‐^13^C DARR NMR spectra is shown in Figure 1 D. The DNP NMR experiments had to be performed at −164 °C in the presence of the biradicyl AmuPOL (Figure 1 D). In spite of the increased NMR linewidths under these conditions, the DNP NMR spectra provided ^13^C chemical shift information for each Trp with the exception of Trp327^7.55^ and Trp173^4.50^, which were ambiguous. The NMR assignment of Trp281^6.48^ and Trp327^7.55^ were confirmed by W281T and W327F mutants studied at −30 °C (see Figure S2 A,B). The chemical shifts of the Trp residues determined at low temperature under DNP conditions were transferred to the −30 °C studies assuming that the sequence of shifts from highest to lowest field did not change. As the Y2R does not undergo melting or other large structural changes in this temperature range, no significant chemical shift changes are expected.

### Native Trp Residues Report the Conformational Dynamics of Y2R

**Figure 2:**
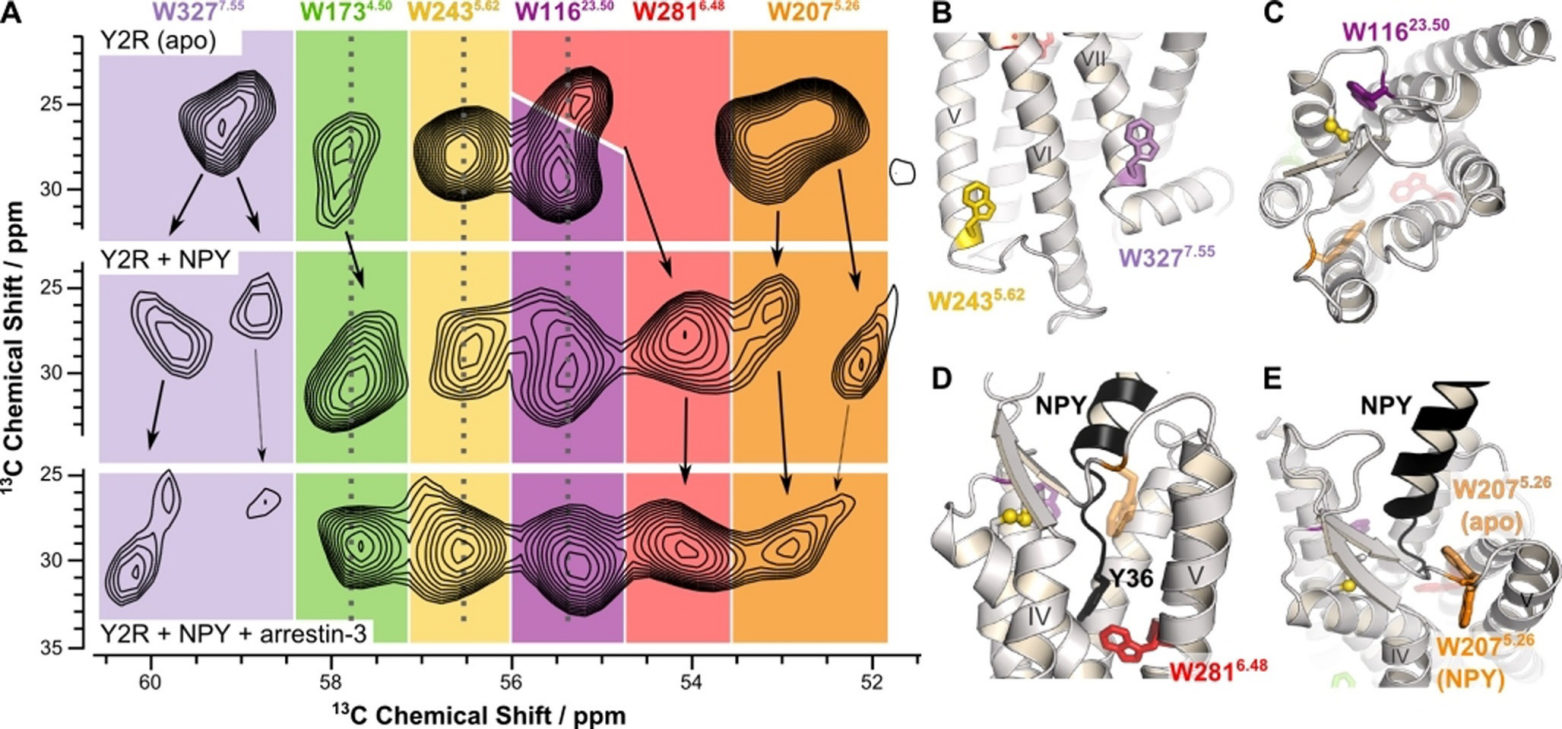
Conformational changes of the native Trp residues of Y2R as observed by ^13^C NMR. A) Cα/Cβ region of ^13^C‐^13^C DARR NMR spectrum of ^13^C‐Trp‐labeled Y2R in DMPC membranes at −30 °C in different functional states: the apo state (top row), the NPY‐bound state (middle row), and the NPY‐ and arrestin‐3 bound state (bottom row). B)–E) Structural details of the Y2R model^[23]^ highlighting the interactions of the Trp residues. B) The sidechains of Trp243^5.62^ and Trp327^7.55^ localized on the intracellular side face the membrane. C) Residue Trp116^23.50^ forms a “cysteine lock” as the indole ring is stacked against the conserved disulfide bridge. D) Y2R is shown in the NPY‐bound state indicating the interaction of the toggle switch residue Trp281^6.48^ with the C‐terminal residue Tyr36 of NPY (black). E) Same model as in D) displaying the conformation of Trp207^5.26^ in the apo and the NPY‐bound states indicating the putative conformational changes between these two states.^[23]^

To record conformational changes of Y2R during activation, the receptor was prepared in three functional states: (*i*) the apo state, (*ii*) the NPY‐bound state (full agonist bound state), and (*iii*) as a ternary complex with NPY and arrestin‐3. Superposition of the corresponding ^13^C NMR spectra revealed characteristic chemical shift changes that can be assigned to dynamic conformational changes of Y2R upon activation (Figure 2 A).

In the apo state, the NMR signals of Trp116^23.50^, Trp243^5.62^, and Trp281^6.48^ are relatively sharp and well separated indicating a single conformation for each of these sites. Trp173^4.50^, Trp207^5.26^, and Trp327^7.55^ display two NMR signals referring to two different conformations. Binding of NPY leads to distinct spectral changes and peak splitting indicative of multiple conformations especially for residues Trp327^7.55^, Trp207^5.26^, and possibly Trp281^6.48^. The differences in chemical shift between resolved NMR signals indicate that the conformations are in slow exchange (the lower correlation time limit is *τ*≈1 ms). The NMR signal of Trp173^4.50^ shows a moderate upfield shift upon agonist binding, whereas the signals of Trp243^5.62^ and Trp116^23.50^ do not change.

The arrestin‐3 bound conformation of the receptor is characterized by one single and sharp NMR signal for four Trp residues suggesting that the receptor has reached a more stable state with lower conformational flexibility. However, for Trp327^7.55^, two weak signals indicative of lowly populated conformations and for Trp207^5.26^ a weak peak shoulder indicates residual conformational diversity. These two residues also show weaker signal intensity suggesting higher dynamics as cross‐polarization is less efficient. Trp327^7.55^ provides an NMR signal shifted downfield by the largest amount from both the apo and the NPY‐bound states. The NMR signal of Trp207^5.26^ was shifted slightly upfield as compared to the apo state and found in between the two signals observed for the NPY‐bound state. The Trp173^4.50^ peak was shifted slightly upfield from the apo state at a similar position as in the NPY‐bound state. The NMR signals of Trp243^5.62^ and Trp116^23.50^ did not change position relative to both the apo and the NPY‐bound states.

### Backbone Motional Amplitude of the Trp Residues in Y2R

**Figure 3:**
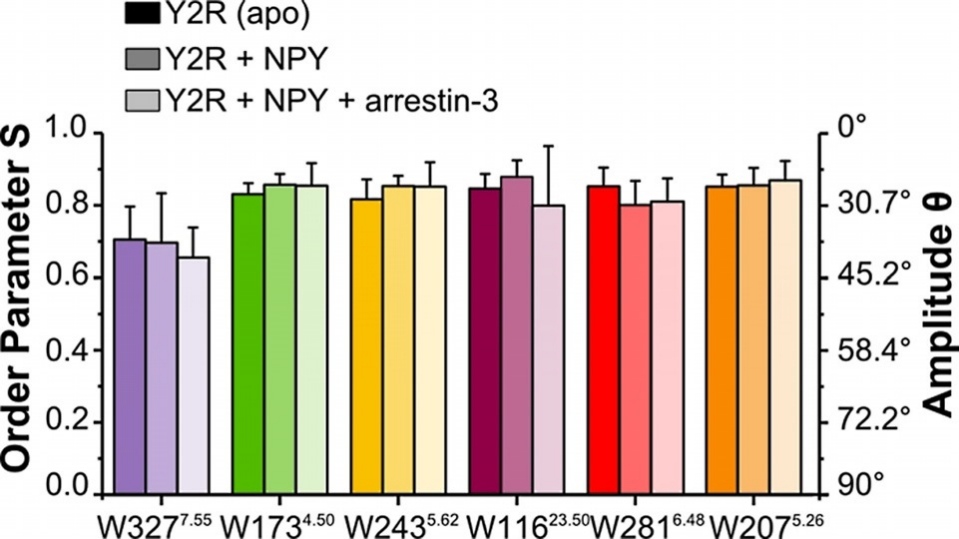
Motional order parameters of the ^1^Hα‐^13^Cα bond vectors of the six Trp residues in Y2R in the apo form, with NPY‐bound, and with NPY and arrestin‐3. Experiments were carried out in DMPC membranes at 5 °C.

To further characterize the conformational dynamics of the Y2R on a fast timescale, molecular order parameter describing the motional amplitude of a bond vector were determined. We used DipShift experiments^[36]^ at 5 °C to measure the motions of the backbone ^1^Hα‐^13^Cα bond vectors of all Trp in Y2R (Figure S3). Order parameters are sensitive to fluctuations with correlation times *τ*<40 μs. Order parameters (*S*, with *S=*1 representing completely rigid, and *S=*0 isotropically mobile segments) measured in each conformational state are shown in Figure 3.

Trp327^7.55^ shows the lowest order parameter of 0.71. The other five Trp residues showed order parameters between 0.82 and 0.85. Addition of NPY and arrestin‐3 did not significantly alter these values based on the errors from at least two independent preparations. For comparison, the order parameter of all backbone sites including the loops and termini of Y2R was previously determined to be 0.69.^[26]^ The Trp residues measured here with site‐resolution are localized in either stable secondary structure motifs, loop structures (Trp207^5.26^), or stabilized by the disulfide bridge (Trp116^23.50^) explaining their somewhat higher values. Order parameters of 0.70 to 0.80 correspond to motional amplitudes in the backbone of ≈35° to 30°.

### Trp Residues Report the Conformational Dynamics of Y2R in Membranes

Many studies have shown that GPCRs exist in several conformations and their biological function is not simply described as an on/off equilibrium of the inactive and active conformations, but follows a complex pattern of highly dynamic structural changes between several substrates.[^17^, ^37^] These dynamic conformational transitions during activation and interaction with intracellular effectors can only be studied in atomistic detail using spectroscopy.[^18^, ^38^] The conformational ensemble of a GPCR in the lipid bilayer is distinctly different from that in detergent micelles^[37]^ necessitating the investigation in planar membranes. With the current solid‐state NMR data, determined in membranes, we describe molecular details of the structural dynamics of Y2R upon binding to its full agonist NPY and in interaction with arrestin‐3‐3A. Currently, there is no crystal structure available for Y2R in any conformational state. In our approach, Y2R produced in CF synthesis with ^13^C‐labeled Trp residues was reconstituted into DMPC membranes allowing to study its activation in a native environment. The probes for the analysis were the Trp backbone atoms, which directly report conformational alterations that are not masked by sidechain or probe rotameric states or any artificial labels.

Trp residues rigidify the protein backbone. The indole ring with its large surface is frequently involved in hydrogen bonds, π stacking, cation‐π, and van der Waals interactions. These properties render Trp residues relatively rigid sites in proteins undergoing only small librations.^[39]^ Three of the six Trp of Y2R do not report significant structural alteration when the molecule goes through the activation cycle. These are localized in the ECL1 (Trp116^23.50^), TM4 (Trp173^4.50^), and at the transition region from TM5 to ICL3 (Trp243^5.62^) and can be considered as located in well‐preserved structural motifs. The other three Trp reflect the dynamic conformational equilibrium reported by pronounced chemical shift changes indicative of structural alterations.

### The Functional and Structural Roles of Trp in Y2R

The NMR results reveal key functions of the Trp residues located in different regions of Y2R. Trp207^5.26^, Trp281^6.48^, and Trp327^7.55^ are subject to conformational flexibility. For these residues, multiple chemical shifts indicative of different receptor conformations were observed in the apo state, which are in slow exchange on the millisecond timescale. Agonist binding is not sufficient to stabilize one active conformation of the molecule. The most well‐known site is Trp^6.48^ described as the “toggle switch” of activation. In all GPCRs investigated so far, TM6 undergoes a large conformational change upon agonist binding representing the most universal feature of these molecules.[^19^, ^37^, ^40^] Trp281^6.48^ is part of the CWxP motif, identified as a SWLP sequence in Y2R and highly conserved in class A receptors. It is located at the bottom of the ligand‐binding pocket in close proximity to the C‐terminal residue Tyr36 of the agonist (Figure 2 D).^[23]^ Mutagenesis experiments showed that Trp281^6.48^ does not significantly contribute to ligand affinity but is required for receptor activation and downstream signaling. Mutagenesis to Thr, His or Tyr strongly reduced the efficacy to activate the G‐protein pathway and to recruit β‐arrestin to the receptor.^[41]^ The Trp281^6.48^ NMR signal most significantly shifts by >1 ppm upfield upon addition of NPY. This is the most prominent chemical shift change of any of the Trp residues upon ligand binding indicating the importance of Trp281^6.48^ in activation. The crystal structure of the A_2A_ receptor revealed that agonist binding causes a pronounced structural alteration at the position of Trp^6.48^, prompting TM6 to tilt and rotate.^[42]^ The importance of Trp^6.48^ in TM6 of rhodopsin has also been described.^[15]^ It is reasonable to hypothesize that TM6 in Y2R undergoes similar conformational changes resulting in the observed chemical shift changes of Trp281^6.48^. No further conformational changes are observed in the arrestin‐3 bound state of Y2R confirming that agonist binding causes the required structural alteration of TM6 for interaction of Y2R with arrestin.

Prominent structural diversity was observed for Trp327^7.55^, located on the intracellular side at the TM7/H8 region, known to undergo large conformational changes in GPCRs upon activation.^[43]^ The position of Trp327^7.55^ is usually occupied by a Phe or another hydrophobic residue in other GPCRs. Trp327^7.55^ is located in the flanking region of TM7, in the apo state most likely oriented towards the membrane and in close proximity to the conserved NPxxY^7.53^ motif. The structure of the (rhod)opsin/arrestin complex indicate that residues Asn^7.57^, Lys^7.58^, and Gln^7.59^ are localized in close proximity to arrestin^[5]^ suggesting that Trp327^7.55^ is highly relevant to report conformational changes upon arrestin binding. Also, in the structure of the neurotensin receptor/arrestin complex,^[6]^ residues Val^7.56^, Ser^7.57^ and Ala^7.58^ interact with the arrestin fingerloop corroborating that Trp327^7.55^ responds sensitively to arrestin binding. Comparison of inactive and active states of class A GPCRs shows that the tip of TM7 undergoes an inward movement upon activation.^[44]^ Thereby, the sidechain of residue 7.55 orients parallel to the backbone of helix 8, most often in a stacking interaction with the sidechain of Lys/Arg^8.51^. This results in a more fixed conformation upon binding of the intracellular binding partner. However, our data show residual conformational flexibility and appreciable dynamics of Trp327^7.55^ in the arrestin‐3‐3A bound state.

Our results suggest an allosteric coupling of Trp327^7.55^ as described for Trp on the intracellular surface of the A_2A_ adenosine receptor.^[14]^ In the apo state of Y2R, a relatively broad NMR signal was detected for Trp327^7.55^ indicating multiple conformations. In the NPY bound state, two clearly separated signals (at ≈59.6 ppm and ≈58.8 ppm) of approximately equal intensity were observed reflecting two distinct conformations. After formation of the ternary complex with arrestin‐3, some conformational as well as the dynamic flexibility remains, however, there is one preferred conformation that is most highly populated. But lower signal intensity indicates a relatively mobile state even at −30 °C. Measurements of the motional amplitude of the Cα‐Hα bond vector of Trp327^7.55^ on a fast time scale indicated that it shows the largest amplitude of motion (≈42°, Figure 3). The side chain of Tyr^7.53^ of the NPxxY motif is repositioned upon activation of GPCRs^[2]^ and an inward movement of Tyr^7.53^ at the intracellular end of TM7 has been observed in the neurotensin receptor.^[45]^ Our results suggest that Trp327^7.55^ of Y2R may also undergo such rearrangement.

The third Trp residue for which a conformational equilibrium is observed is Trp207^5.26^, located in the ECL2 of Y2R. A broad NMR peak in the apo state suggests Trp207^5.26^ to be structurally flexible. Hydrophobic contacts with the agonist are believed to constrain NPY at an angle of approximately 45° relative to the membrane normal.^[23]^ Thr204^5.23^ and Glu205^5.24^ in close proximity to Trp207^5.26^ significantly contribute to NPY binding energy.^[23]^ Thus, it was expected that Trp207^5.26^ is influenced by the agonist. In the apo state, Trp207^5.26^ points towards the inside of Y2R. It is involved in interactions with TM5, TM6 and the antiparallel strand of the extracellular β‐sheet. Upon NPY binding, Trp207^5.26^ shows two NMR signals suggesting two different conformations. In the NPY bound state, Trp207^5.26^ is sandwiched between TM4 and TM5 at least in one conformation (Figure 2 E). The conformational flexibility of Trp207^5.26^ in the agonist bound state reflected in the two peaks in the DARR NMR spectrum was significantly abrogated upon arrestin‐3 binding showing one dominating NMR signal of lower intensity with residual molecular dynamics.

Little structural dynamics was found for Trp173^4.50^. Although highly conserved (94 %) within the rhodopsin‐like GPCR family, only a slight upfield shift of the NMR signal of Trp173^4.50^ was detected upon NPY and arrestin‐3 binding.

Trp243^5.62^ is located at the end of TM5 pointing towards the membrane.^[23]^ No functional importance has been assigned to this site. It is not conserved within class A GPCRs or the NPY receptor family. While it is membrane exposed, there is no indication for conformational flexibility. In the model of Y2R,^[23]^ Trp243^5.62^ is in a one‐helix‐turn distance to Tyr239^5.58^ which shows 75 % conservation in class A GPCRs. Upon activation, Tyr^5.58^ forms hydrogen bonds to Tyr^7.53^ of the NPxxY^7.53^ motif as well as to Arg^3.50^ of the DRY/ERY motif, considered as “water lock”.^[44]^ Comparison of inactive and active states of GPCR structures shows that despite the formation of new interactions, the relative positions of Tyr^5.58^ and residue 5.62 do not change upon binding of the intracellular binding partner explaining the lack of any response of Trp243^5.62^ to arrestin‐3 binding.

Finally, an interesting role in the architecture of Y2R is found for Trp116^23.50^, located in the conserved WxxG motif of ECL1. It is expected to pack with its indole ring against the conserved disulfide bridge.^[39]^ Examining the available crystal structures, a Cys lock motif is observed in many class A GPCRs and appears to stabilize the conformation. In some cases even two or three Trp are in the vicinity of the disulfide bridge and might stabilize the conformation of this region (e.g. in the histamine H1, muscarinic and serotonin receptors). Furthermore, comparison of the inactive and active states of rhodopsin and β2‐adrenergic receptor shows that this motif is independent of the activation state. This was also reflected in the ^13^C NMR spectra, where the peak of Trp116^23.50^ showed no chemical shift alterations.

In contrast to the dynamic transitions between the individual conformations of the Y2R during the activation cycle seen as exchange broadening in ^13^C NMR spectra reflecting conformational transitions, explicit measurements of the amplitudes of the fast fluctuations of the Trp residues did not produce significant differences (Figure 3). This suggests that the fast thermal fluctuations reflecting the packing of the receptor interior and its interaction with the lipid molecules is not influenced by the dynamic transitions between the conformational states on the lower microsecond and sub‐microsecond time scale as probed in the DipShift experiment. Thus, DipShift probes the conformational flexibility in the individual wells of a given state, while the exchange‐broadening of the NMR signals reports the biologically relevant conformational transitions.

## Conclusion

**Figure 4:**
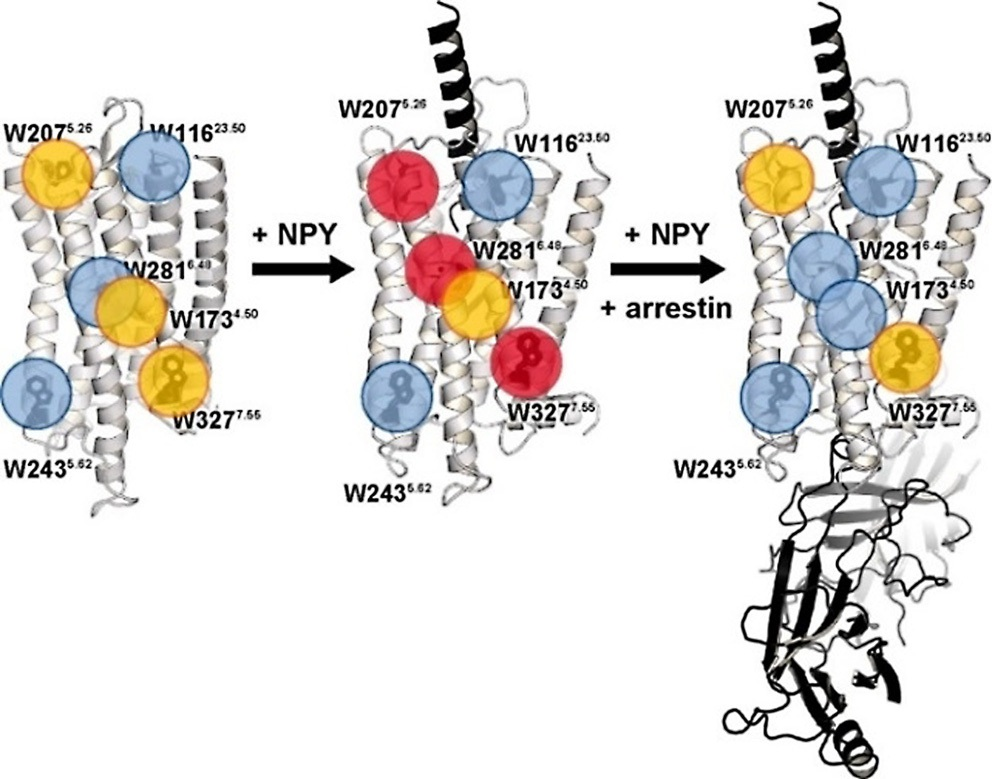
Summary of the structural dynamics of Y2R visualizing the conformational equilibria of the Y2R in the apo state (left), in the NPY‐bound state (middle) and in complex with NPY as well as arrestin‐3‐3A (right). Trp residues with low, intermediate, and high conformational flexibility are highlighted by blue, yellow, and red circles, respectively. The structures on the left side and in the middle refer to the published structural models,^[23]^ the complex shown on the right represents just a visualization of arrestin binding for illustration.

We describe the conformational states of Y2R during activation by analyzing NMR chemical shift information from six natural Trp residues. Trp have been used as probes to study the dynamic conformational alterations of GPCRs before.[^13^, ^14^, ^33^] However, instead of the protein backbone, usually the NMR signals of the indole ring were exploited. Analyzing the receptor backbone sites provides the most direct approach to observing the structural response of the molecule to agonist and intracellular effector binding.^[15]^ Using solid‐state NMR spectroscopy, GPCRs are studied in a relatively native bilayer environment.^[46]^ As in other class A GPCRs, the activation of Y2R is mediated by a series of dynamic conformational changes which are reported here by the native Trp residues. Our results on the structural dynamics of the Y2R are summarized in Figure 4. The measurements revealed a conformational equilibrium of Y2R in the apo and agonist bound states. This flexibility is reduced upon arrestin binding which largely stabilizes one predominant conformation that is different from the apo and agonist bound states. However, some residual conformational flexibility and molecular dynamics of Y2R in that state remains. Out of the well‐described molecular switches triggered upon GPCR activation, the toggle switch residue Trp281^6.48^ showed a pronounced response to agonist and arrestin‐3 binding in agreement with an outward movement of TM6 upon activation. Furthermore, residues Trp207^5.26^ and Trp327^7.55^ at the TM7/H8 boundary close to the NPxxY motif report further structural alterations during activation. For the opsin/arrestin complex,^[5]^ a similar structural architecture as in the β2‐adrenergic receptor/Gs complex has been identified^[47]^ involving the interaction of the finger loop of arrestin with the NPxxY motif also observed in Y2R. In the GPCR‐arrestin complexes, a multimodal interaction network that also includes arrestin interactions with the membrane has been identified.[^6^, ^7^, ^8^] The intriguing membrane contribution to GPCR/arrestin complex formation specifically calls for investigating the GPCR/arrestin interaction in membrane environment as carried out in the current study. Finally, Trp116^23.50^‐Cys lock formation was identified for Y2R. The combination of cell‐free labeling, investigation of the conformational states of the backbone sites by solid‐state NMR in membranes, and the investigation of the interaction of GPCRs with specific lipids represents an attractive option to gain biological insight into the function of GPCRs, for which no crystal structures are available.

## Conflict of interest

The authors declare no conflict of interest.

## Supporting information

As a service to our authors and readers, this journal provides supporting information supplied by the authors. Such materials are peer reviewed and may be re‐organized for online delivery, but are not copy‐edited or typeset. Technical support issues arising from supporting information (other than missing files) should be addressed to the authors.

Supplementary
