# Supplementary material for: The Conformational Equilibrium of the Neuropeptide Y2 Receptor in Bilayer Membranes
Source: Angew Chem Int Ed Engl. 2020 Sep 30;59(52):23854–61. doi: 10.1002/anie.202006075 (PMC7736470; doi:10.1002/anie.202006075)
Supplement: Supplementary file 1 — Supplementary [file ANIE-59-23854-s001.pdf]

Angewandte Chemie
Eine Zeitschrift der Gesellschaft Deutscher Chemiker

Supporting Information

The Conformational Equilibrium of the Neuropeptide Y2 Receptor in Bilayer Membranes

*Ulrike Krug, Anika Gloge, Peter Schmidt, Johanna Becker-Baldus, Frank Bernhard, Anette Kaiser, Cindy Montag, Marcel Gauglitz, Sergey A. Vishnivetskiy, Vsevolod V. Gurevich, Annette G. Beck-Sickinger, Clemens Glaubitz, and Daniel Huster**

anie_202006075_sm_miscellaneous_information.pdf

METHODS

DNA preparation for CF expression. For CF expression, the pIVEX2.3d vector (biotechrabbit GmbH, Hennigsdorf, Germany) was modified in that the *Nco*I site was mutated from an ATG to a GCG codon using the PfuUltra II fusion HS DNA polymerase (Agilent, Waldbronn/Germany). According to a standard PCR protocol, the gene of Y2R was cloned into the modified pIVEX2.3d vector via the *Nde*I and *Xma*I (NEB, Frankfurt am Main/Germany) restriction sites using Phusion® High Fidelity DNA Polymerase (NEB, Frankfurt am Main/Germany). The final DNA sequence coded for an N-terminal SER-tag[1] which was inserted between Met1 and Gly2, the Y2R gene containing cysteine mutations (i.e. Cys58$^{1.40}$Ala, Cys103$^{2.57}$Ser, Cys151$^{3.53}$Ser, Cys272$^{6.39}$Ala, Cys316$^{7.44}$Ala, Cys342Ala,[2]) and a C-terminal ProGlyGlyGlySerHis$_6$ sequence. Large-scale DNA preparation was accomplished with the QIAGEN Plasmid Midi Kit (Qiagen, Hilden/Germany).

CF expression. The protocol for CF expression of Y2R is based on previous work.[3] The preparation scheme of the CF expression is shown in Table S1. The amino acids were dissolved in 100 mM Hepes at a pH of 7.4 (Asn, Asp, Cys, Gln, Glu, Trp, and Tyr) or in ddH$_2$O (all other amino acids). All amino acid stock solutions were adjusted to a final pH between 7.0 and 7.4. The concentration was 100 mM, except for Tyr (20 mM) and Trp (50 mM). The amino acids were combined in an amino acid mix, in an RWCMDE mix (to provide an additional amount of the amino acids more prone to degradation) and in an AILSTV (as the six most abundant amino acids of the protein sequence). During the experiments, it turned out to be advantageous to add Cys separately as several freezing/thawing cycles lead to precipitation of the stock solution presumably caused by formation of cystine. For selective isotopic-labeling, amino acids were replaced in the amino acid mixes by the isotopically-labeled analogs. All chemicals were dissolved in ddH$_2$O to concentrations of the stock solutions as indicated in Table S1. The nucleotides were dissolved to a concentration of 240 mM (CTP, GTP, and UTP) and 360 mM (ATP) and mixed in a ratio of 1:1:1:1 (v/v). The pH of the phosphoenolpyruvate solution was adjusted to pH ~7 by potassium hydroxide. The stock solution of the S30 extract buffer contained 10 mM Tris, 14 mM magnesium acetate, 0.6 mM potassium acetate, and 0.5 mM DTT (pH 8.2). For each experiment, a fresh 50× cOmplete (EDTA-free Protease Inhibitor Cocktail) solution (Sigma Aldrich, Taufkirchen /Germany) was prepared by dissolving 1 tablet in 1 ml ddH$_2$O.

All solutions (except folinic acid) were kept on ice during preparation. S12 extract was added to the reaction mix with a final concentration of up to 40% (v/v). CF expression was carried out with the reaction mix (usually 2 ml) filled in a dialysis bag (MWCO 12-14 kDa, ZelluTrans/Roth, Karlsruhe/Germany) that was placed in a weighing dish filled with the feeding mix (usually 34 ml). The weighing dish was covered by a second one and parafilm sealed the reaction chamber. The reaction was incubated for 24 h at 34°C and 80 rpm.

Preparation of *E. coli* cell extract for CF expression. The S12 extract used in CF expression was prepared from *E. coli* Rosetta(DE3). Cells were cultivated in 25 ml of 2×YTPG medium with 34 µg/ml chloramphenicol. 2×YTPG medium consisted of 16 g/l tryptone, 10 g/l yeast extract, 5 g/l NaCl, 22 mM $NaH_2PO_4 \cdot 2\,H_2O$, 40 mM Na_2HPO_4, and 1 % (w/v) glucose. The cells were transferred in a 200 ml overnight culture, then in a 200 ml fermentation starter culture, and finally at an OD600 of 1.5 with a dilution of 1:20 into the fermenter (with a total volume of cell medium of 3 l). Cells were cultivated at 37°C, at pH 7, with an air flow of 8 l/min, and a stirring rate of 600 to 800 rpm. Cells were harvested on ice after they had reached an OD600 of ~3.5 to 4.0. They were centrifuged at 5,000 *g* and 4°C for 15 min. The pellet was resuspended twice in S30 extract buffer (10 mM Tris-acetate, 14 mM magnesium acetate, 60 mM potassium acetate, pH 8.2) followed by centrifugation at 4,000 *g*, and 4°C for 15 min. Finally, the pellet was resuspended in 10 ml of S30 extract buffer per 8 g of cells and the suspension stored in Falcon tubes on ice overnight in the coldroom. Before cell lysis, 1× cOmplete™ (EDTA-free Protease Inhibitor Cocktail) and 1 mM DTT were added to the solution. Cell lysis was accomplished by an APV 2000 homogenizer. After centrifugation at 12,000 *g* and 4°C for 30 min, the supernatant was incubated at 30°C and 150 rpm for 2 h. The supernatant was placed into a dialysis bag (MWCO 12-14 kDa, Zellu Trans Roth) and dialyzed against a 40-fold excess of S30 extract buffer supplemented with 1 ml/l 2-

WILEY-VCH

Mercaptoethanol in two steps at 4°C. The extract was snap frozen in liquid nitrogen in aliquots of 800 µl and stored at -80°C until usage.

Purification and reconstitution of Y2R. After 24 h of CF expression, precipitated Y2R was solubilized by addition of 9 ml 50 mM sodium phosphate buffer (pH 6.5), 15 mM SDS, and 50 mM DTT per 1 ml reaction mix. The excess of DTT was removed in a two-step dialysis against the same buffer without DTT. The protein solution was adjusted to pH 8 by NaOH and the denatured protein purified by immobilized metal affinity chromatography using a 5 ml HisTrap™ HP column (GE Healthcare, Germany).[4] The buffers used in chromatography consisted of 50 mM sodium phosphate and 15 mM SDS, with pH 8 for the equilibration buffer and pH 4 for the elution buffer. Protein yields of 0.7 to 1.4 mg per 1 ml reaction volume were determined by UV absorption (NanoDrop, Spectrophotometer ND-1000, peqlab Biotechnologie GmbH). Protein purity was verified by SDS PAGE analysis. Y2R was reconstituted in two steps as described:[4] first, the SDS concentration was reduced by dialysis and the disulfide bond was formed by addition of a glutathione redox system (0.2 mM reduced glutathione, 0.1 mM oxidized glutathione) to a carefully degassed buffer containing 50 mM sodium phosphate, 2 mM SDS, 1 mM EDTA (pH 8.5). Subsequently, the receptor was concentrated by addition of 25 % (w/v) PEG20,000 to the same buffer. Second, the receptor was incorporated into preformed lipid bicelles of 1,2-dimyristol-*sn*-glycerol-3-phosphocholine (DMPC or chain-deuterated DMPC-d_{54}) and 1,2-diheptanoyl-*sn*-glycero-3-phosphocholine (DHPC) in 50 mM sodium phosphate (pH 7) by fast temperature changes from 42°C to 0°C. The molar ratio was 1:200:800 of receptor:DMPC:DHPC. Lipids were purchased from Avanti Polar Lipids via Merck (Darmstadt, Germany). After removal of residual detergent using BioBeads SM2 (Bio-Rad, München/Germany), the samples were centrifuged at 21,500 × *g*.[4] For experiments in the presence of NPY, Y2R reconstituted in lipid bicelles was incubated with the peptide ligand in a molar ratio of 1:1.5 overnight at room temperature. Finally, the samples were centrifuged and the pellets loaded into the MAS rotors.

Peptide synthesis. NPY was synthesized by automated solid phase peptide synthesis following the Fmoc/*tert*-butyl strategy as described before.[5] In brief, the peptide sequence is built up from C- to N-terminus in 15 µmol scale on Rink amide resin by repetitive cycles of Nα-Fmoc deprotection, activation, and coupling of the next amino acid in a Syro II peptide synthesizer (MultiSynTech, Witten/Germany). All reactive side chains carried protection groups that were stable under the conditions of the Fmoc cleavage. Fmoc was cleaved by successive treatment with 40% piperidine and 20% piperidine in *N,N*-dimethylformamide (DMF) for 3 and 10 minutes, respectively. The amino acids were conjugated in a double coupling procedure (2 x 45 min) with 8 eq. Nα-Fmoc-amino acid in situ activated with 8 eq. ethyl 2-cyano-2-(hydroxyimino)acetate and 8 eq. diisopropylcarbodiimide in DMF.

For fluorescence labeling, For fluorescence labeling, 5/6 carboxy-tetramethylrhodamine (TAMRA) was coupled to the free N-terminus of [Ahx_{5-24}]NPY as described for related NPY variants.[6] Briefly, 3 eq. TAMRA, 3 eq. HATU (O-(7-azabenzotriazole-1-yl)-1,1,3,3-tetramethyluronium-hexafluorophosphate), and 3 eq. DIPEA (N,N-diisopropylethylamine) were dissolved in 200 µL DMF and incubated with the resin-bound peptide for 3 h in the dark.

The peptides were cleaved off the resins by using trifluoroacetic acid/H_2O/triisopropyl silane (90/5/5, v/v/v) for 2 hours, which simultaneously removes all remaining side chain protection groups. The peptides were precipitated using ice-cold diethyl ether. The identity of the peptides was confirmed by MALDI-TOF (matrix-assisted laser desorption/ionization-time of flight) mass spectrometry (Ultraflex III MALDI-TOF/TOF; Bruker, Billerica/USA), and the peptides were purified to 95% by reversed-phase high performance liquid chromatography as reported previously.[5]

Functional characterization of Y2R. A fluorescence polarization assay was carried out to determine the ligand binding capacity of reconstituted Y2R obtained from CF expression.[4,7,8] The receptor was reconstituted in DMPC/DHPC bicelles at a molar ratio of 1:600:2400 in 50 mM sodium phosphate buffer pH 7. Subsequently, various concentrations of Y2R were incubated with 50 nM TAMRA-[Ahx_{5-24}]NPY overnight.

WILEY-VCH

This peptide variant is a reduced-size Y2R-specific agonist, which activates Y2R-expressing HEK293 with nanomolar potency (pEC50 8.87 ± 0.17 vs 10.35 ± 0.08 of NPY). Measurements were carried out on a FluoroMax-2 (JOBIN YVON) spectrometer in a 10 mm quartz cuvette at 20°C with linear polarized light, an excitation wavelength of 549 nm, an emission wavelength range of 574 to 578 nm, and 90° detection angle. Three independent measurements were carried out in triplicate. A sigmoidal dose-response curve was used to fit the data with the Origin software.

Preparation of Y2R + NPY + arrestin-3 sample. For NMR experiments of Y2R in the presence of arrestin, the phosphorylation-independent variant of *bos taurus* arrestin-3[9] was added. This variant contained the 3A mutation (Ile397Ala, Val398Ala, Phe399Ala) as described previously.[9] The modified arrestin-3 was prepared as described.[10] Briefly, the protein was expressed in *E. coli* Rosetta(DE3) or *E. coli* NiCo21(DE3) cells in LB medium at 26°C and 150 rpm. Expression was induced by addition of IPTG to a final concentration of 35 µM at an OD600 of ~1.0 to 1.5. A multistep cell lysis included the addition of lysozyme (Roth, Karlsruhe/Germany), freezing at -80°C, sonication, incubation with 8 mM $MgCl_2$ plus DNase (Sigma, Taufkirchen/Germany) and several centrifugation steps. The protein was precipitated by the addition of ammonium sulfate to a final concentration of 2.4 M, pelleted, and dissolved in column buffer. The following chromatography steps included purification on a heparin-Sepharose column, Q- and SP-Sepharose columns (GE Healthcare). The purification steps were validated by SDS-PAGE and Western blot.

For incubation with Y2R, arrestin-3 was dialyzed in two steps against 50 mM sodium phosphate (pH 7), 1 mM EDTA, and 200 mM NaCl. The ternary complex was formed by incubation of Y2R reconstituted in DMPC/DHPC bicelles with NPY (twofold molar excess) and arrestin-3 (threefold molar excess) overnight at 4°C. The solution was centrifuged and the pellet loaded into the MAS rotor.

Binding of arrestin-3 to Y2R was verified by a pull-down assay. To this end, the proteins were separated from the lipids by chloroform/methanol precipitation and the pellet resolved in SDS sample buffer. The samples were analyzed via SDS-PAGE and Western Blot. The F431 rabbit polyclonal antibody[11] was used for detection of arrestin-3.

NMR spectroscopy. Membrane samples containing up to 6 mg of isotopically labeled and reconstituted Y2R were subjected to NMR spectroscopy. The samples were filled into 3.2 mm MAS rotors. Bruker Avance III 600 MHz and Avance Neo 700 MHz NMR spectrometer using a triple resonance MAS probes with a 3.2 mm spinning module were used. Homonuclear ^{13}C-^{13}C DARR correlation experiments[12] were performed at a temperature of -30°C and MAS frequencies of 11 or 12 kHz with a mixing time of 10 ms and a CP contact time of 2 ms. The DipShift pulse sequence[13] was applied at an MAS frequency of 5 kHz with the 90° pulses adjusted to 4 µs for both channels. CP contact times of 200 µs, 700 µs or 2 ms were used for the excitation of the ^{13}C nuclei. For decoupling, the spinal sequence (heteronuclear ^{1}H-^{13}C decoupling[14]) and the FSLG sequence (homonuclear ^{1}H-^{1}H decoupling[15]) at an radiofrequency field strength of ~80 kHz were applied. To ensure objective data evaluation, spectral intensity was integrated over defined spectral regions based on the peaks of the ^{13}C-^{13}C DARR NMR spectra. The order parameters from the DipShift experiments resulted from the ratio of the motionally averaged dipolar coupling strength and the rigid limit of the dipolar coupling.[16] The dipolar coupling strength was derived from best fit of numerically simulated dipolar dephasing curves fitted to the experimental dephasing curves measured over one rotor period (9 increments). At least two independent samples were prepared for each conformational state of Y2R. The molecular order parameters were converted into amplitude information using the formula $S = \cos(\theta)(1 + \cos(\theta))/2$.[17]

DNP experiments. Samples of Y2R labeled with ^{13}C-Trp and either ^{15}N-Lys, ^{15}N-Gly, ^{15}N-Leu, ^{15}N-Pro, ^{15}N-Ser, or ^{15}N-Met and reconstituted into DMPC-d_{54} membranes were pelleted via centrifugation and shipped on dry ice to Frankfurt/Main (Germany) within two days. The wet pellets were incubated with 50-100 µl of 20 mM AmuPOL in Glycerol-d_8:D_2O:H_2O (30:60:10) at 4°C overnight.[18] The supernatant was removed and the soaked pellet was packed into 3.2 mm ZrO_2-MAS rotors. DNP experiments were recorded using a Bruker 400 MHz DNP

system consisting of a 400 MHz WB Avance II NMR spectrometer and a 263 GHz Gyrotron as a microwave source equipped with a 3.2 mm HCN low temperature MAS probe. All experiments were recorded at an MAS frequency of 8 kHz at a temperature of around -164°C. The microwave power at the probe was 10 W. Spectra were referenced indirectly to DSS using the right signal of the glycerol-d_8 at 64.78 ppm. All experiments were recorded with 100 kHz high power decoupling, CP contact times of 1 ms, a recycle delay of 1.2-1.8 s (depending in the ^{1}H T_1 times of the samples), a 4 ms specific NCO transfer and 20 ms PDSD mixing for the CX step. Typically 40,000 and 180,000 scans were recorded for each sample.

WILEY-VCH

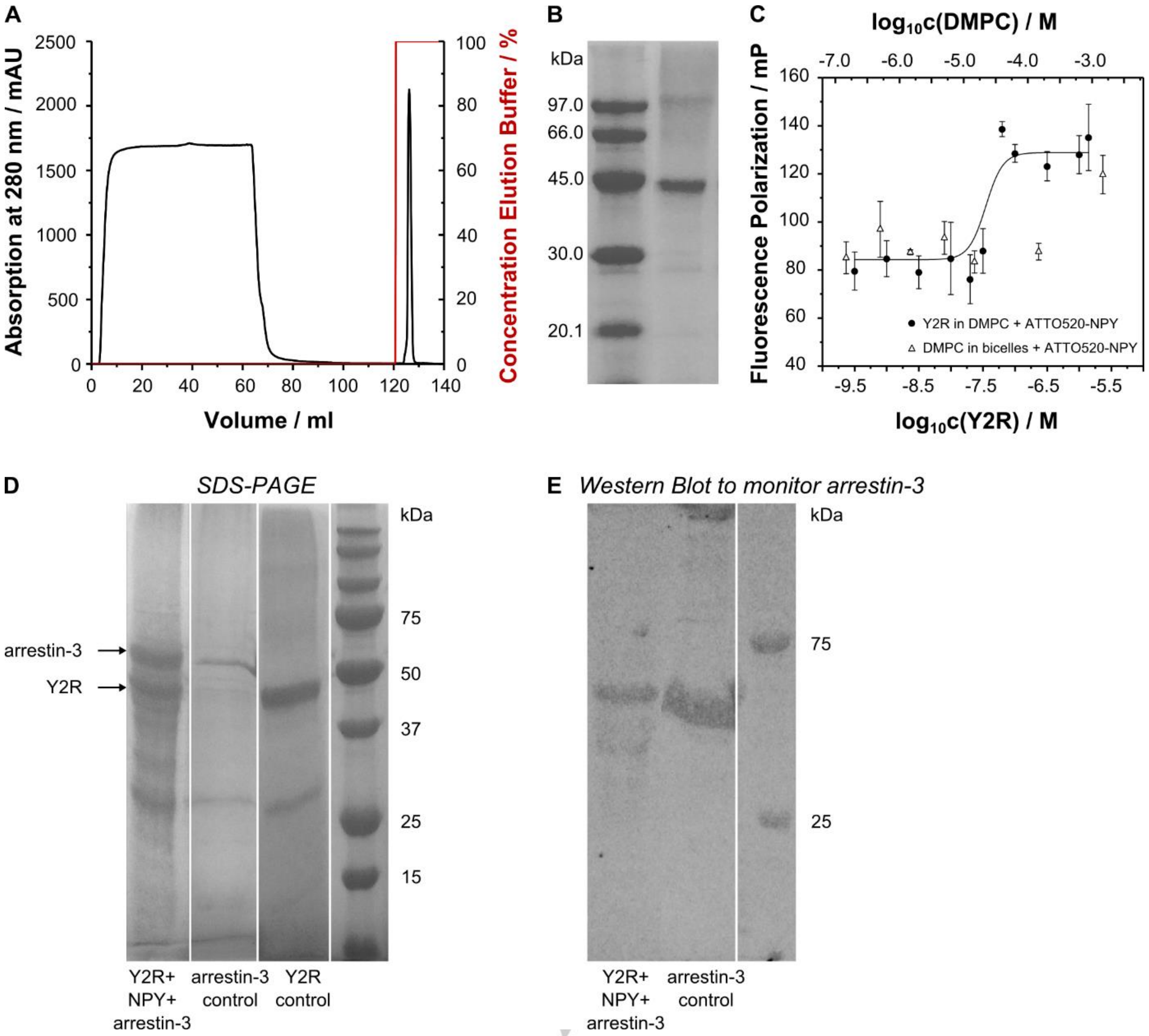

Figure S1. Summary of analytical and pharmacological data on protein preparation of Y2R by cell-free expression and formation of the ternary complex of Y2R+NPY+arrestin-3. Precipitated Y2R obtained by cell-free expression was solubilized in SDS, purified by immobilized metal affinity chromatography and eluted by a pH shift. Panel A) reproduces a typical chromatogram of the purification B) SDS-PAGE of purified Y2R (Y2R shows a prominent band with a molecular weight of about 44 kDa). C) Fluorescence polarization assay to monitor ligand binding to the reconstituted Y2R.[6] Varying Y2R concentrations were incubated with 50 nM of TAMRA-labeled [Ahx_{5-24}]NPY. Only weak ligand binding to empty lipid bicelles (in the absence of Y2R) is observed. The Y_2 receptor binds [Ahx_{5-24}]NPY with similar nanomolar affinity (K_i = 13 nM) in cellular membranes.[19] D) SDS-PAGE showing the ternary complex of Y2R+NPY+arrestin-3 as visualized after a pull-down assay. E) Western Blot analysis to monitor the presence of arrestin-3 in the sample Y2R+NPY+arrestin-3. Arrestin-3 was detected with the F431 rabbit polyclonal antibody.[10] The control shows purified protein.

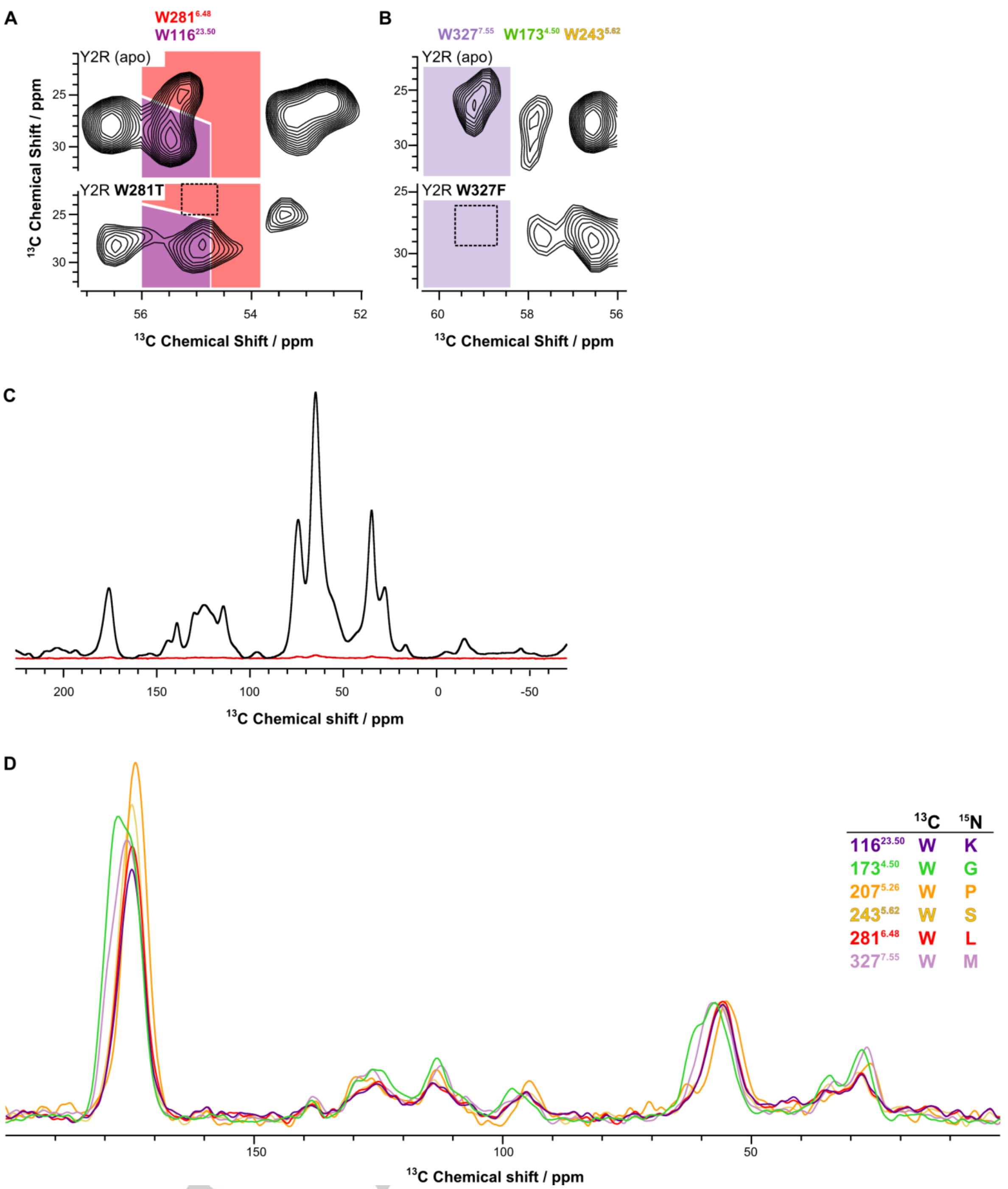

Figure S2. NMR Assignment of Trp281$^{6.48}$ and DNP-enhanced MAS NMR of ^{13}C-W/^{15}N-X-labeled Y2R. A-B) Cα/Cβ region of ^{13}C-^{13}C DARR correlation experiments of the apo state of ^{13}C-Trp-labeled Y2R along with A) the Y2R mutant W281T, and B) the Y2R mutant W327F recorded in DMPC membranes at -30°C. C) Cross polarization experiment of ^{13}C-W/^{15}N-K labeled Y2R. Experiments were performed at a temperature of -164°C, with an MAS of 8 kHz, and with 128 transients. 20 mM AmuPOL were added to the sample. Using DNP (black) a gain in sensitivity by a factor of 70 was achieved (compare black and red spectra). D) 1D NCOCX ^{13}C NMR spectra of six ^{13}C-W/^{15}N-X-labeled Y2R variants indicated by different colors. The labeling scheme indicating the ^{13}C-labeled Trp and its ^{15}N-labeled successor amino acid is given.

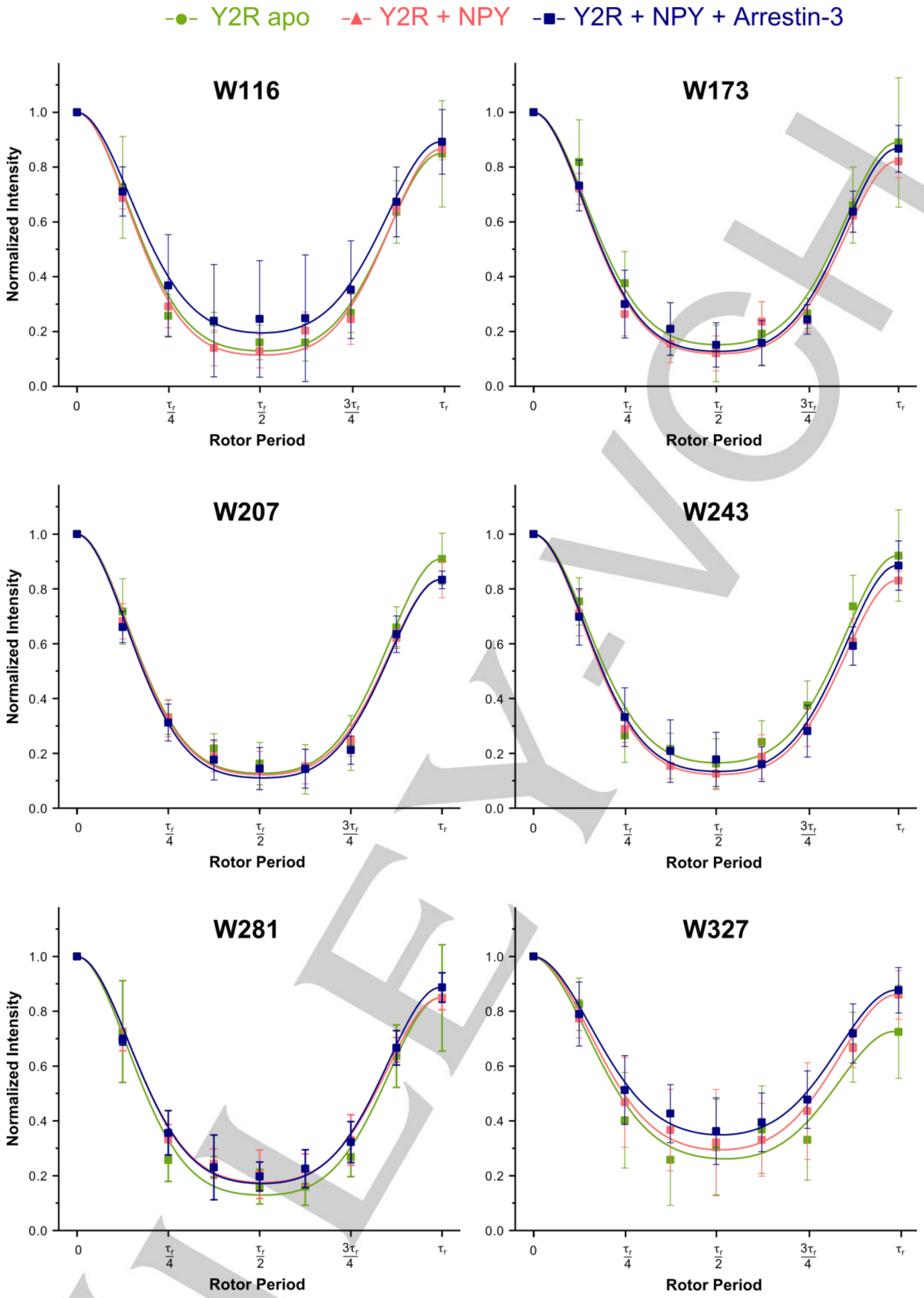

Figure S3. Representative ^{1}H-^{13}C dipolar dephasing curves determined from DipShift experiments of the six Trp residues of Y2R in DMPC membranes at 5°C. Data points are determined from at least two independent preparations of the Y2R in the various states. Solid lines represent the best fit simulations to determine the motional averaged dipolar coupling.

Table S1 (Related to Figure 1 and Figure 2): Preparation sheet for cell-free expression of $^{13}C/^{15}N$-Trp-labeled Y2R. Compounds were mixed in a master mix split into a feeding and a reaction mix. The feeding mix and the reaction mix are supplemented with further reactants to a final volume of 17 ml and 1 ml, respectively.

Compound	Supplier	Concentration of Stock Solution	Master Mix		
ddH_2O			438 µl		
HEPES buffer (pH 8.4)	Roth	2.5 M	660 µl		
Magnesium acetate	Roth	2 M	93.2 µl		
Potassium acetate	Riedel-de Haen	4 M	511.5 µl		
PEG 8000	Roth	40 %	900 µl		
NaN_3	Merck	10 %	90 µl		
Folinic acid calcium salt hydrate	Merck	20 mg/ml	90 µl		
Dithiothreitol	Roth	500 mM	72 µl		
ATP/CTP/GTP/UTP-mix (1:1:1:1 mix of 360 mM ATP, 240 mM CTP, 240 mM GTP, 240 mM UTP)	AppliChem, Merck	75×	240 µl		
cOmplete™, EDTA-free Protease Inhibitor Cocktail	Merck	50×	360 µl		
Phosphoenolpyruvate-KOH	AppliChem	1 M	360 µl		
Lithium potassium acetyl phosphate	Merck	1 M	360 µl		
amino acid-mix					
Amino acid-mix (w/o Cys, w/o Trp)	Merck	4.5 mM each amino acid	1925 µl		
Cys	Merck	100 mM	87.5 µl		
$^{13}C/^{15}N$-Trp	Merck	50 mM	175 µl		
$RC^{13C15N}WMDE$					
RMDE	Merck	25 mM each amino acid	719 µl		
Cys	Merck	100 mM	180 µl		
$^{13}C/^{15}N$-Trp	Merck	50 mM	180 µl		
AILSTV	Merck	16.7 mM each amino acid	1078 µl		
		Total volume	8519 µl	**Feeding Mix**	**Reaction Mix**
		Ratio to split Master Mix		*16.05*	*0.95*
Master Mix as prepared above				8043 µl	476 µl
S30 extract buffer				5950 µl	
Amino acid-mix (w/o Cys, w/o Trp)	Merck	4.5 mM each amino acid		1925 µl	
Cys	Merck	100 mM		87.5 µl	
$^{13}C/^{15}N$-Trp	Merck	50 mM		175 µl	
Pyruvate kinase	Merck	10 mg/ml			4 µl
tRNA from *E. coli* MRE 600	Merck	40 mg/ml			12.5 µl
T7 RNA Polymerase	Life Technologies	200 U/µl			30 µl
RiboLock RNase Inhibitor	Life Technologies	40 U/µl			7.5 µl
DNA (SER-Y2R×pIVEX2.3d)	QIAGEN Midi Prep Kit	939.7 ng/µl			27.7 µl
S12 cell extract		100 %			400 µl
ddH_2O				820.5 µl	42.3 µl
**Total (µL)**				17000 µl	1000 µl

References

[1] S. Haberstock, C. Roos, Y. Hoevels, V. Dötsch, G. Schnapp, A. Pautsch, F. Bernhard, *Protein Expr. Purif.* **2012**, *82*, 308.

[2] K. Witte, A. Kaiser, P. Schmidt, V. Splith, L. Thomas, S. Berndt, D. Huster, A. G. Beck-Sickinger, *Biol. Chem.* **2013**, *394*, 1045.

[3] D. Schwarz, F. Junge, F. Durst, N. Frölich, B. Schneider, S. Reckel, S. Sobhanifar, V. Dötsch, F. Bernhard, *Nat. Protoc.* **2007**, *2*, 2945.

[4] P. Schmidt, B. J. Bender, A. Kaiser, K. Gulati, H. A. Scheidt, H. E. Hamm, J. Meiler, A. G. Beck-Sickinger, D. Huster, *Front. Mol. Biosci.* **2018**, *4*, 100.

[5] A. Kaiser, P. Müller, T. Zellmann, H. A. Scheidt, L. Thomas, M. Bosse, R. Meier, J. Meiler, D. Huster, A. G. Beck-Sickinger et al., *Angew. Chem. Int. Ed. Engl.* **2015**, *54*, 7446.

[6] V. Mäde, K. Bellmann-Sickert, A. Kaiser, J. Meiler, A. G. Beck-Sickinger. *ChemMedChem*. **2014**, *9*, 2463.

[7] S. Schrottke, A. Kaiser, G. Vortmeier, S. Els-Heindl, D. Worm, M. Bosse, P. Schmidt, H. A. Scheidt, A. G. Beck-Sickinger, D. Huster, *Sci. Rep.* **2017**, *7*, 46128.

[8] B. J. Bender, G. Vortmeier, S. Ernicke, M. Bosse, A. Kaiser, S. Els-Heindl, U. Krug, A. Beck-Sickinger, J. Meiler, D. Huster, *Structure* **2019**, *27*, 537-544.

[9] J. Celver, S. A. Vishnivetskiy, C. Chavkin, V. V. Gurevich, *J. Biol. Chem.* **2002**, *277*, 9043.

[10] S. A. Vishnivetskiy, X. Zhan, Q. Chen, T. M. Iverson, V. V. Gurevich, *Curr. Protoc. Pharmacol.* **2014**, *1*, 2-11.

[11] X. Song, S. A. Vishnivetskiy, J. Seo, J. Chen, E. V. Gurevich, V. V. Gurevich, *Neuroscience* **2011**, *174*, 37.

[12] a) K. Takegoshi, S. Nakamura, T. Terao, *Chem. Phys. Lett.* **2001**, *344*, 631; b) K. Takegoshi, S. Nakamura, T. Terao, *J. Chem. Phys.* **2003**, *118*, 2325.

[13] M. G. Munowitz, R. G. Griffin, G. Bodenhausen, T. H. Huang, *J. Am. Chem. Soc.* **1981**, *103*, 2529.

[14] G. de Paëpe, A. Lesage, L. Emsley, *J. Chem. Phys.* **2003**, *119*, 4833.

[15] A. Bielecki, A. C. Kolbert, M. H. Levitt, *Chem. Phys. Lett.* **1989**, *155*, 341.

[16] D. Huster, L. Xiao, M. Hong, *Biochemistry* **2001**, *40*, 7662.

[17] A. G. Palmer III, J. Williams, A. McDermott. *J. Phys. Chem.* **1996**, *100*, 13293.

[18] J. Becker-Baldus, C. Glaubitz, *eMagRes* **2018**, *7*, 79

[19] S. Østergaard, J. Kofoed, J. F. Paulsson, K. G. Madsen, R. Jorgensen, B. S. Wulff. *J. Med. Chem.* **2018**, *61*, 10519.

WILEY-VCH
